# Youth Perspectives on the Recommended Age of Mobile Phone Adoption: Survey Study

**DOI:** 10.2196/40704

**Published:** 2022-10-31

**Authors:** Aliah Richter, Victoria Adkins, Ellen Selkie

**Affiliations:** 1 Child Health Evaluation and Research Center University of Michigan Ann Arbor, MI United States

**Keywords:** adolescent, youth, child, mobile phone, technology, media, phone use, phone ownership, parental guidance, parenting, cell phone, smartphone

## Abstract

**Background:**

Despite increasing prevalence of phone ownership in early adolescence, there is a deficit of evidence-based guidance on the appropriate time to provide youth their first phone.

**Objective:**

This survey study explored age recommendations for phone ownership among a diverse panel of youths, as their experiences are an important contribution to the development of ownership guidelines.

**Methods:**

Participants were recruited from MyVoice, a national panel of over 765 youth (14 to 24 years old) who respond to weekly SMS text message–based surveys. Questions were distributed between January 24 2018, and March 20, 2018. Inductive qualitative analysis was used to identify major themes among youths’ open-ended responses.

**Results:**

In all, 469 youth (mean age 18.8 years; female: 299/469, 63.8%; White race: 332/468, 70.8%) responded. On average, respondents obtained their first phone at 12.2 years of age. Most participants (325/459, 71.1%) stated they received their first phone out of necessity rather than for entertainment or social reasons. Youth recommended that early adolescents receive their first phone between 12 and 13 years of age primarily for reasons of necessity (146/448, 32.6%).

**Conclusions:**

According to the participants, phones supported safety and independence by allowing communication with parents and participation in activities. Youth-serving professionals and parents can incorporate these youth perspectives into shared decision-making about phone ownership among families. This can include discussions about essential features, safety, or phone use, as well as maturity and responsibility milestones, which were all key considerations reported by participants in the survey.

## Introduction

Mobile phone adoption in the United States is starting in late childhood and early adolescence; currently, 53% of children have a smartphone by age 11 [1]. Later in adolescence, mobile phone use remains high, with over 95% of teens ages 13 to 17 years having access to a cell phone [2]. In addition, over 90% of teens report using their phones to pass the time, connect with others (84%), and learn new things (83%) [3,4].

The introduction and frequent use of mobile technology during adolescence comes at a critical stage of cognitive, emotional, and social development. Between the ages of 10 and 25 years, youths are in a transitory state between childhood and adulthood. According to Erikson’s theory of psychosocial development, adolescents are primarily concerned with the task of identity formation [5]. More specifically, they are learning about their values, desires, and future roles [5]. In addition, adolescents are confronted with other milestones, including learning how to navigate intimate relationships and gain social connectivity [5,6]. During this stage, adolescents spend more time with peers and gain independence from their caregivers [7]. This period is sometimes referred to as separation-individuation, a developmental term that has been used to define the phenomenon of adolescents needing less support and approval from their caregivers while seeking approval from peers and finding new social networks to help develop individual concepts of self-esteem and identity [8,9].

As adolescents’ exposure to new digital technologies increases, they are progressively experiencing these milestones online, with online communication being used as a tool offering access to friendships and other relationships, as well as increasing cohesion and connectedness between peers and school [10]. Mobile phone adoption poses as a major concern for parents or caregivers and youth-serving professionals [11]. However, sources that explore mobile phone adoption, such as the American Academy of Pediatrics, only offer general suggestions, like encouraging family discussions about the type and quantity of media consumed and monitoring impacts of phone use on sleep, homework, and family time [12,13]. Official guidelines on the appropriate age to get a phone have yet to be established.

Evidence-based recommendations for mobile phone ownership are needed to establish best practices for parents and youth-serving professionals and alleviate apprehension towards mobile phone adoption. Despite the ubiquity of mobile phone use among adolescents and the personal stake that adolescents have in guidelines about mobile phone adoption, the youth voice regarding this topic is understudied. Exploring youth experiences with mobile phone ownership may benefit parent involvement and guidance, as well as aid in the development of rules and expectations that promote safe use, are developmentally appropriate, and account for social and cultural pressures. Therefore, the purpose of this study is to explore age recommendations for mobile phone ownership among a diverse text message–based panel of youths.

## Methods

### Participants

Participants were members of MyVoice, a national panel of over 765 adolescents and young adults (14 to 24 years old) who respond to weekly surveys delivered through text messages [14]. MyVoice recruits participants through advertisements on social media platforms (Facebook, Instagram) and in person at community events. Youth consented to receive surveys (parental permission was waived), and youth could indicate if they wanted to skip or stop participation at any time through a text message. Participants received a US $5 gift card for completing a demographic survey after enrollment and US $1 for each survey completed afterward [14]. Demographic data, including age, gender, race, ethnicity, and education, were self-reported during enrollment using validated questions from the Youth Risk Behavior Survey [15].

### Ethics Approval

This study was approved by the University of Michigan Institutional Review Board (#HUM00119982).

### Survey

Our survey consisted of 4 questions which were delivered via SMS text message to participants between January 24, 2018, and March 20, 2018. The questions were authored by several youth medicine providers and researchers with specialty knowledge in this research area. Participants had 1 week to answer the survey questions, all questions were open ended, and participants had the option to skip questions they did not want to answer. We asked the following four questions: (1) “How old were you when you got your first cell phone?” (2) “Did you ask for your first cell phone or did you get it without asking?” (3) “Why did you get your first cell phone?” (4) “What do you think is the right age for someone to get their first cell phone and why?”

### Analysis

Two study team members (LR and VA) reviewed the answers to the questions of the first 100 participants and used inductive qualitative analysis to independently develop a codebook based on their responses. The final codebook was formed by merging the independently developed codebooks and by arriving at a mutual agreement on final definitions. Each question had its own set of codes that could be applied, and multiple codes could be assigned to each participant response. The responses to each question were coded in rounds of 100; since there were a limited number of responses, the first 100 were recoded as well. After each round, the team met to discuss discrepancies. Interrater reliability over all rounds of coding was between 77% and 95% for each question. A third study team member (ES) broke a total of 5 discrepancies that could not be agreed on. Descriptive statistics for code frequencies were calculated using Microsoft Excel. This coding and analysis scheme has been used in previous MyVoice studies [16-19].

## Results

### Overview

From a total of 765 MyVoice participants, 469 respondents completed at least 1 text message–based survey question about mobile phone ownership (61.3% response rate). Specifically, 465 respondents provided the age at which they got their first cell phone (60.8%), 465 respondents discussed if they asked for a phone or received it without asking (60.8% response rate), 459 respondents described why they got their first cell phone (60% response rate), and 448 respondents shared their recommendations for mobile phone ownership (58.6% response rate). Respondents had an average age of 18.8 (SD 3.01) years, and 63.8% (299/469) identified as being female and 70.8% (332/469) as being White, non-Hispanic. Demographic characteristics of these 469 respondents are described in Table 1.

**Table 1:** Demographic characteristics of the study sample.

Characteristic		Respondents (n=469)	Nonrespondents (n=80)
**Age, mean (SD)**		18.79 (3.0)	17.73 (2.7)
	14-17 years, n (%)	174 (37.1)	45 (56.2)
	18-21 years, n (%)	184 (39.2)	27 (33.7)
	22-24 years, n (%)	109 (23.2)	8 (10)
**Gender, n (%)**			
	Female	299 (63.8)	35 (43.7)
	Male	139 (29.6)	41 (51.2)
	Other gender	12 (2.5)	0 (0)
	Nonbinary	10 (2.1)	0 (0)
	Transgender FTM^a^	8 (1.7)	3 (3.7)
	Transgender MTF^b^	0 (0)	1 (1.2)
**Race, n (%)**			
	White	332 (70.8)	66 (82.5)
	Asian	51 (10.8)	3 (3.7)
	African American	45 (9.5)	12 (15)
	Multiracial	29 (6.1)	0 (0)
	Other race	7 (1.4)	4 (5)
	American Indian	3 (0.6)	5 (6.2)
	Pacific Islander	1 (0.2)	1 (1.2)
**Ethnicity, n (%)**			
	Non-Hispanic	430 (91.6)	67 (83.7)
	Hispanic	38 (8.1)	13 (16.2)
**Education level, n (%)**			
	Some college	155 (33.0)	27 (33.7)
	Some high school	149 (31.7)	40 (50)
	Bachelor's degree	66 (14.0)	3 (3.7)
	High school graduate	44 (9.3)	3 (3.7)
	Associate degree	17 (3.6)	1 (1.2)
	8th grade or less	15 (3.2)	3 (3.7)
	Some graduate school	14 (2.9)	1 (1.2)
	Master's degree	4 (0.8)	1 (1.2)
	Some graduate training beyond a master's degree	2 (0.4)	0 (0)
	Some vocational/technical training	1 (0.2)	0 (0)
	Completed vocational/technical training	1 (0.2)	1 (1.2)
	Doctoral degree	0 (0)	0 (0)
**School free or reduced lunch, n (%)**			
	No	339 (72.2)	57 (71.2)
	Yes	124 (26.4)	22 (27.5)

^a^FTM: female to male.

^b^MTF: male to female.

### Question 1: How Old Were You When You Got Your First Cell Phone?

The average reported age at which respondents obtained their first phone was 12.2 (SD 2.01) years, with answers ranging from 4 to 18 years old.

### Question 2: Did You Ask for Your First Cell Phone or Did You Get It Without Asking?

When asked how they obtained their phone, 59.1% (275/465) of participants reported asking for it while 34.2% (159/465) received their phone without asking. These data are summarized in Table 2. The remainder of participants did not answer if they asked for their phone or got it without asking; instead, they provided other answers such as making a mutual decision with their parents to get their first phone or buying it on their own. Some participants (57/465, 12.2%) qualified their responses by describing the circumstances of their phone ownership, including that they received their phone from a nonparental source—such as an extended family member (5/465, 1.1%), their phone was inherited (7/465, 1.5%), they received their phone as a gift (6/465, 1.3%), or they got it out of convenience (6/465, 1.3%).

**Table 2:** Youth responses to Q1 and Q2 about when they got their first phone and if they asked for it.

Question			Value (N=465)
**Q^a^1: How old were you when you got your first cell phone?**			
	Age (years), mean (SD)	12.2 (2.01)	
	Age (years), response range (min-max)	4-18	
**Q2: Did you ask for your first cell phone or did you get it without asking?, n (%)**			
	Asked	275 (59.1)	
	Did not ask	159 (34.2)	

^a^Q: question.

### Question 3. Why Did You Get Your First Cell Phone?

#### Note on Overlapping Themes

Two distinct categories emerged from participant responses in answers to both question 3 and question 4: (1) phone function, which refers to identifying the purpose of their phone usage; and (2) environmental context, which refers to providing perceptions about phone ownership within their current social environments. The following themes were identified across these categories in which participants described their mobile device use: necessity, maturity as a requirement, socialization, environmental context, and maturity as a result. Although these categories generally corresponded to the survey questions, there was overlap in the participants’ responses that applied to multiple questions. Participant responses, themes, and representative quotes are described in Table 3.

#### Phone Function

The first category of responses focused on the functionality of phones; that is, the intended purpose(s) of phone use for each individual.

#### Necessity

Necessity for communication was cited by 71.1% (325/459) of respondents as their primary reason for getting a phone. Respondents obtained their phone to contact their parents (162/459, 35.4%), have it in case of emergency (83/459, 18.2%), and to participate in afterschool activities (68/459, 14.9%). One participant explained they got their first phone “To call my parents after school because I stayed late for sports and other things a lot.”

#### Maturity as a Requirement

Respondents (59/459, 12.9%) also associated phone ownership with specific life experiences or as a part of growing up and becoming more mature. For example, respondents described reaching a certain point in their education (33/459, 7.2%) or having greater independence and going out more on their own without parental supervision (16/459, 3.5%) as contexts for obtaining their first phone. One respondent said they got their first phone because of the following:

…i [sic] was going to middle school, and getting more independent, so i [sic] needed communication. It was also normal for most middle schoolers to have phones.

#### Socialization

Wanting to stay in touch with friends and family members was the primary reason why 12.9% (59/459) of participants got their first cell phone. A respondent reasoned, “I wanted to be able to talk to my friends without having to use my dad’s phone.”

#### Environmental Context

The second category of responses focused on environmental context or, more specifically, observations about phone ownership within surrounding social climates. Respondents (31/459, 6.8%) attributed their desire for phone ownership to the phone ownership of others; that is, via social comparison or just a general feeling of needing to fit in. These participants typically had responses like “Because everyone else had one.”

**Table 3:** Youth responses to question 3 about reasons for getting their first phone (N=459).

Subtheme by theme				Respondents, n (%)	Representative quote
**Overall theme: functionality**					
	**Subtheme: necessity**		325 (71.1)		N/A^a^
		Contacting parents	162 (35.4)		“So my parents could communicate with me” (F^b^; age 14 y, got phone at 12 y)
		Safety	83 (18.2)		“because i got lost once, and my parents wanted me to have one for security purposes” (F; age 16 y, got phone at 8.5 y)
		Afterschool activities	68 (14.9)		“Because I had activities after school and needed a phone to call my parents to pick me up afterwards” (F; age 24 y, got phone at 13 y)
	**Subtheme: maturity as a requirement**		59 (12.9)		N/A
		Education	33 (7.2)		“At that point I was going into middle school and I had more need to be able to contact my parents” (M^c^; age 18 y, got phone at 13 y)
		Independence	16 (3.5)		“…I [sic] was going to middle school, and getting more independent, so i needed communication. It was also normal for most middle schoolers to have phones.” (F; age 16 y, got phone at 11 y)
		Socialization	59 (12.9)		“I wanted to more easily communicate with my friends…” (M; age 24 y, got phone at 14 y)
Overall theme: environmental context				31 (6.8)	“Because everyone else had one.” (No gender indicated; age 16 y, got phone at 9.5 y)

^a^N/A: not applicable.

^b^F: female.

^c^M: male.

### Question 4. What Do You Think Is the Right Age for Someone To Get Their First Cell Phone and Why?

Overall, 79.9% (354/448) of respondents suggested a specific age or age range in their phone ownership recommendations. The average recommended age was 12.7 (SD 1.66) years. Participant responses, themes, and representative quotes are described in Table 4.

#### Necessity

Respondents (146/448, 32.6%) discussed the condition of necessity in their recommendations for phone ownership. When asked for their age recommendations, one respondent replied as follows:

Around 13 or when entering middle school, children become more independent and do things without their parents there. It's important to be able to stay in touch with your parents.

Another respondent commented as follows:

As soon as they need it honestly. If a child is frequently out and about (eg community centers/clubs, friends, paper routes), has a situation where they need to contact their parent, has to wait after class, etc, then the actual age doesn't matter much…

#### Maturity as a Requirement

In their recommendations, 10.8% (48/448) of respondents felt individuals tend to experience maturity at the specific ages they suggested and incorporated this reasoning into their recommendations. One respondent recommended, “[age] 14. Then they are mature enough for it.” Conversely, 13.6% (60/448) of respondents felt an individual’s readiness for phone ownership was contingent on individual internal factors such as achieved independence (38/448, 8.6%) and maturity (22/448, 4.9%). These respondents felt individuals experience independence and responsibility at different ages, so they did not offer specific age recommendations for phone ownership. Rather, they cited proxy indicators of maturity. For instance, one participant put it as follows:

I think it depends on the person. If someone is living a more independent life going out with friends alone or traveling on their own they should get a phone to communicate.

#### Socialization

Additionally, 5.4% (24/448) of respondents recommended getting a phone to socialize with friends and manage relationships. One participant posited that the right time to get a phone was “maybe 8th grade? it’s an important thing to have for social connection and most of their peers will have a phone in middle school.”

#### Environmental Context

Reflection on changing trends regarding the initial age of phone ownership was included in 3.6% (16/448) of respondents’ recommendations. For example, one respondent commented, “I think the age [to get a first phone] keeps getting younger and younger. Maybe 12 years old.” Other age recommendations that expanded on this observation included the following:

I think now it’s more necessary for kids to have them earlier. I think they shouldn’t have a smart phone until their [sic] at least 14 though…

15. Cellphones are a vital part of today’s communication.

Respondents (3/448, 0.7%) even felt adolescents would be at a disadvantage if they did not own a phone while their peers did:

I think that it's younger than when I received one, because the technology has advanced and withholding one would make the child stand out from their peers.

#### Maturity as a Result

Further reflections described a bidirectional association between phone ownership and maturity. Although participants’ responses showed that mobile phone ownership may be dependent on “maturity-indicating” milestones, such as reaching a certain grade level or spending more time away from home, they also presented phone ownership as its own significant adolescent milestone that could foster maturity. Specifically, 2.5% (11/448) of respondents felt phone ownership could provide adolescents the opportunity to learn about responsibility, and subsequently, become more mature. When asked for their recommended age for phone ownership, one participant replied as follows:

I would say late middle school to the start of high school. This is the time when young people need to learn more about freedom and responsibility as well as when they may need it to communicate with their parents.

Similar responses included the following:

2-13. Kids start to become more independent at that age, and a cell phone helps establish that independence.

The best age would be 12-14, because this is the best time to teach them the value of responsibility.

**Table 4:** Youth responses to Q4 about mobile phone ownership recommendations (N=448).

Subtheme by theme and question			Value	Representative quote
**Q4^a^: What do you think is the right age for someone to get their first cell phone and why?**				
	Suggested a specific age or age range		354 (79.9)	N/A^b^
	Recommended age (years), mean (SD)		12.7 (1.6)	N/A
	Recommended age (years), response range (min-max)		7-20	N/A
**Qualitative responses, n (%)**				
	Subtheme: necessity		146 (33)	“If a child is frequently out and about (eg community centers/clubs, friends, paper routes), has a situation where they need to contact their parent, has to wait after class, etc, then the actual age doesn't matter much…” (M^c^; age 20 y, got phone at 12 y)
	**Subtheme: maturity as a requirement**		60 (13.6)	
		Conditional independence	38 (8.6)	“I think it depends on the person. If someone is living a more independent life going out with friends alone or traveling on their own they should get a phone to communicate.” (M; age 14 y, got phone at 12 y)
		Conditional responsibility	22 (5)	“when they are mature enough to use it responsibly, so i think it depends per person.” (F^d^; age 20 y, got phone at 12 y)
		Responsibility	48 (10.8)	“[age] 14. Then they are mature enough for it.” (No gender indicated; age 17 y, got phone at 14 y)
	Subtheme: socialization		24 (5.4)	“maybe 8th grade? it’s an important thing to have for social connection and most of their peers will have a phone in middle school.” (F; age 23 y, got phone at 11 y)
	**Subtheme: environmental context**			
		Observation	16 (3.6)	“I think the age [to get a first phone] keeps getting younger and younger. Maybe 12 years old.” (F; age 24 y, got phone at 14 y)
		Social disadvantage	3 (0.7)	“I think that it's younger than when I received one, because the technology has advanced and withholding one would make the child stand out from their peers.” (Nonbinary; age 18 y, got phone at 11 y)
	**Subtheme: maturity as a result**			
		Learning opportunity	11 (2.5)	“The best age would be 12-14, because this is the best time to teach them the value of responsibility.” (M; age 14 y, got phone at 12 y)

^a^Q4: question 4.

^b^N/A: not applicable.

^c^M: male.

^d^F: female.

### Additional Findings

Although the survey questions did not explicitly ask about possible negative aspects of early mobile phone ownership, a small proportion of youth (34/448, 7.7%) brought up concerns in their phone ownership recommendations. Participant responses, themes, and representative quotes are described in Table 5. The highest cited concern was that of distraction (13/448, 2.9%), where participants voiced concerns about phones producing interference with daily activities and even overreliance. One participant commented in their age recommendation: “14. Kids are inundated in a life reliant on their phones if they're introduced any earlier.” Another concern included unrestricted internet access (4/448, 0.9%), where a respondent recommended the following:

12, but it’s important that they don’t start with a smart phone. Being safe on the internet requires maturity - kids shouldn’t have access to the web on their phones until they’re ~15.

Addiction (4/448, 0.9%) was another concern found in phone ownership recommendations. One recommendation mentioned that “…apps are addictive, unregulated substances, and shouldn't be given freely to young children.”

Participants also discussed concerns regarding illicit activities such as sexting (4/448, 0.9%). In their age recommendation, a participant explained as follows:

The problem is that, nowadays, the phones do so much more than my Nokia did - I don’t think 11 would be okay for an iPhone, for example. There have to be more conversations with kids nowadays with what is and is not okay to do online - look at the amount of kids who get in trouble with nudes. It’s troubling because you want to teach them to safely use the internet, but I think we all know we can’t just give them full access, so where is the line?

**Table 5:** Concerns of mobile phone use as an additional theme (N=448).

Theme or subtheme			Respondents n (%)		Representative quote
**Theme: concerns**			34 (7.7)		N/A^a^
	Distraction	13 (2.9)		“14. Kids are inundated in a life reliant on their phones if they're introduced any earlier.” (M^b^; age 19 y, got phone at 11 y)	
	Unrestricted internet access	4 (0.9)		“12, but it’s important that they don’t start with a smart phone. Being safe on the internet requires maturity - kids shouldn’t have access to the web on their phones until they’re ~15.” (M; age 17 y, got phone at 12.5 y)	
	Addiction	4 (0.9)		“…apps are addictive, unregulated substances, and shouldn't be given freely to young children.” (nonbinary; age 18 y, got phone at 11 y)	

^a^N/A: not applicable.

^b^M: male.

## Discussion

### Principal Results

For this study, youth participants responded to text message–based survey questions about mobile phone ownership through MyVoice. With exception of a few studies examining early adolescent smartphone ownership, adolescent perspectives remain largely unexplored in research [20,21]. This is the first study of youths’ experiences with phone ownership using a large national sample.

For the majority of our respondents, receiving their first cellphone coincided with starting middle school, a significant milestone where many youths are using public transportation on their own, staying after school for activities, and hanging out with friends. It is not surprising that the main reason for getting a phone was for necessity and specifically for contacting parents. Participants’ recommendations for phone ownership also involved these adolescent milestones.

It is important to note that some participants believed phone ownership stands as its own milestone, specifically as a mechanism to learn about responsibility. This is an intriguing concept that parents and youth-serving professionals should explore. During adolescence, youth are experiencing the process of separation-individuation, where they are learning how to navigate social situations with less support from caregivers and establishing their own identity and self-esteem; they are also seeking out peer approval as opposed to parental approval [9]. However, studies have shown that while youth may have less need for parental support, parental involvement is still important [8,9]. Prior research shows that youth who feel they can tell their caregiver of their activities and friendships and feel they can negotiate setting limits are more likely to tell their caregiver when there is a safety concern and are less likely to engage in risky behaviors [22-25]. More research is needed on the impact of phones in the parent-child relationship, but there is a possibility that phones could serve as tools that allow youths to maintain their autonomy while remaining connected to their parents.

The desire for socialization was cited by participants as a reason for getting their first phone, and their recommendations reflected the importance of staying in touch with friends and family. This is an indication that phones can serve as tools for managing and strengthening the quality of peer and parent-youth relationships. The nurturing of these relationships is a crucial task of adolescence, as peer connections help establish separation from parents and encourage independence while simultaneously maintaining a line of communication with caregivers [7].

Although this was not a focus of the study, some participants voiced concerns about mobile phone ownership in their age recommendations, including distraction and overreliance, unrestricted internet access, addiction, and illicit activities. Our report showed a small proportion of youths shared concerns about phone ownership. In other studies, more youths have raised concerns, such as worrying about how much time they spend on their phone [4]. However, since our respondents were not explicitly asked about negative aspects or concerns of phone ownership, our report may not be reflective of our entire sample’s concerns. Based on previous research, parents or caregivers share similar concerns about their children spending too much time on their phones and how phones impact their ability to focus and allow access to inappropriate online content [4,11,26].

Other major concerns that were brought up in previous research are primarily shared by parents or caregivers but were not found in our youth respondents’ recommendations; they include stranger interaction, impact on reputation, and advertisers’ use of data [27]. These parent or caregiver and youth concerns can be included in conversations about mobile phone ownership and shared decision-making. Additionally, as youths and parents are entering the phone market, technology companies producing phones and associated apps can further adjust the experience to maximize safety and provide appropriate features depending on the need and maturity level of the child.

### Comparison With Prior Work

Our findings are concordant with other studies that suggest positive outcomes associated with digital technology use, such as strengthened relationships, promotion of safety, preservation of youth freedom, and greater self-esteem [28-32], as well as with research that presents negative outcomes associated with mobile phone usage, including problematic mobile phone and internet usage, online harassment, and cyberbullying involvement [33-39]. Our study complements other studies’ findings and adds the unique perspective that phones could foster maturity and feelings of responsibility among youths.

### Limitations

MyVoice served as a useful online platform for the recruitment and engagement of youth participants. However, the scope of our survey questions was not as broad as that of traditional qualitative survey questions since respondents had limited space to discuss their experiences (given the character limitations placed on text messages from some phone carriers). Our ability to seek additional context and clarification was also inhibited due to the automated nature of our survey questions. No research was available to guide the formation of our survey questions. Although we posed questions about “mobile phone” ownership, the lack of specification for certain types of phones and, subsequently, consideration of different features, such as internet access on smart phones, could have skewed participants’ age recommendations. Although the MyVoice sample spans the entirety of the United States, the generalizability of our findings may also be limited since MyVoice is not specifically designed to be nationally representative in terms of race, ethnicity, or other demographics. However, the sample is still racially and ethnically diverse and inclusive of multiple sexual and gender minorities. The average age of respondents at the time of survey completion (18.8 years) was older than the reported age at which they received their first phone (12.2 years), which introduces the potential issue of recall bias. Future studies could further explore perspectives from youths at the actual age of phone acquisition. In addition, since our study was conducted prior to the COVID-19 pandemic, youth perspectives on mobile phone ownership might have changed in the context of the pandemic. Further research is needed to characterize any new youth perspectives.

### Conclusions

Our study presents valuable implications about youth mobile phone ownership. Parents report a desire for guidance to navigate their children’s phone ownership and experience, but there remains a lack of evidence-based recommendations [12,21]. In addition, youth viewpoints about their own technology use are understudied in research despite the fact that mobile phone ownership often occurs during adolescence. Our study’s examination of youth perspectives provides insights to the motivations for mobile phone ownership, informs parent decisions about when to introduce a phone, and may promote safe use and behavior. Families and youth-serving professionals can use our findings to facilitate shared decision-making about mobile phone ownership with youths. Shared decision-making allows parents and youths to mutually negotiate rules and expectations about their phone ownership that promote their health and well-being, independence, and safety.
